# Who will steer the antibiotic stewardSHIP ship when I am 80?

**DOI:** 10.1017/ash.2024.489

**Published:** 2025-01-17

**Authors:** Debra A. Goff

**Affiliations:** The Ohio State University Wexner Medical Center, The Ohio State University College of Pharmacy, Columbus, OH, USA

This summer, I saw the Rolling Stones perform outdoors in Chicago. Mick Jagger strutted around with moves that would make a 20-year-old jealous. The music icon is 80. All three music legends played their hearts out, dressed in sparkling fuchsia pink, green, and blue sequin tops. I saw them perform in the 1980s with the same energy and passion. After 60 years in the music industry, it was extra special to see them performing at age 80 and that “they still have got it.”

After the concert, I reflected on my 35-year career in infectious diseases (ID) and antibiotic stewardship. Colleagues ask me, “When are you going to retire?” My first thought is, “Do I ever retire from the fight against antibiotic resistance and global implementation of antibiotic stewardship programs (ASP)? I think not!” I frequently say that doing antibiotic stewardship is like being on a ship that is on a long journey. The captain steers the ship, but all hands are on deck, to make the journey. What happens to the future of ASP if mid-career ID stewards keep exiting the ship (ie, hospitals), in droves, to work for pharmaceutical and diagnostic companies? Will there be any ID stewards with greater than 10–15 years of experience left to steer the ship, when I disembark?

Why are ID pharmacists leaving direct patient care positions at a time when more ID experts with stewardship experience are needed? When ID pharmacists tell me they are leaving their hospital position, I ask them why. There are these three common themes: lack of work–life balance, no dedicated time to conduct ASP research, and not feeling appreciated for their efforts to improve patient care. Some of the responses include: “I am on-service providing direct patient care 12 months of the year, while also precepting students and residents in addition to doing didactic lectures and ASP research. My research is done on my own time. There are no opportunities for advancement in my career. After obtaining funding for an ASP study, I learned I could not get any protected research time like my physician collaborators.” ID pharmacists who recently left clinical pharmacy hospital practices described what led to their breaking points.^1^

Almost 50% of the ID-PGY2 residents I trained have left their hospital positions mid-career to join pharmaceutical and diagnostic companies. Now they write research protocols for new antimicrobials and diagnostic tests and find key opinion leaders who can execute studies. They tell me they have a better work–life balance, much higher salaries, and opportunities for advancement and they feel appreciated. If ID pharmacists continue to jump ship, can ID physicians steer the stewardship ship alone? It is unlikely. Over 80% of the counties within all 50 states do not have an ID physician. The existing shortfall among ID MDs, combined with dwindling physician interest in ID specialization due to low salaries and work–life balance concerns, is very problematic for the future of ASP.^2^ I talk regularly with physician friends who retired from full-time positions and return as a part-time professor emeritus. They still enjoy seeing patients, but they want more time to travel and enjoy life while still in good health. They want a better work–life balance.

Equally concerning is the recent journal series on antimicrobial resistance written by experts speaking at the World Health Assembly.^3^ The authors state that “with limited evidence on the impact of ASP on antimicrobial resistance in low-middle-income countries (LMICs), LMICs are recommended to prioritize funding for infection control, WASH (water, sanitation, and hygiene), and vaccinations. When I finished reading this paper, I felt the antibiotic stewardship ship just hit an iceberg and was on its way to sinking. Imagine what the appropriate use compliance rates for new antibiotics will be if antibiotics are released in LMICs without any antibiotic stewardship oversight? A 2022 paper provided some insights.^4^ The authors report the rapid development of resistance to ceftazidime-avibactam after its unchecked use in private-sector South African hospitals without stewardship. Suggesting that LMICs abandon antibiotic stewardship due to limited funding is a mistake. After decades of infection control education and funding, hospitals still do not have 100% hand hygiene compliance, but that does not mean we should abandon infection control education and funding.

## Individual strategies to prevent burnout

I am frequently asked why I have not experienced “burnout.” After working full-time for 6 years in internal medicine, I had my first child and made a career change as the first ID clinical pharmacist at The Ohio State University Wexner Medical Center. The new position was full-time, but I negotiated to work part-time (50%), and I have worked my entire ID career as a part-time clinical pharmacist. Working part-time meant I had to decline salary and some opportunities and be OK with it. I made wise decisions; I have a productive career. Why am I still steering an ASP ship when I could get off and retire? Meaningful ASP collaborations that affect patient care keep me on the ship. Publishing my research led to opportunities to work in hospitals across 36 countries developing and implementing multidisciplinary ASP. Every trip is an opportunity to learn from others. I always have my camera to photograph what I see and a journal to document what I learn from the people I work with. Providing opportunities for the next generation of ID pharmacist leaders to work and publish with healthcare providers in LMICs is crucial. Although my new ASP experiences are valued, without protected time for this work can contribute to burnout.^1^ I continue doing antibiotic stewardship for our children’s children. In our grandson’s first year of life, he received 6 courses of antibiotics for recurrent otitis media. I could not help but think he may have an antibiotic-resistant pathogen and could lose his hearing. After tympanocentesis and a withdrawal from day care, he has not had another episode of otitis media in 2 years.

## Collective strategies to prevent burnout

The key to longevity in ID ASP is to provide meaningful work and to have strong relationships with a network of people who help to support you and inspire you.^5^ Set boundaries and learn how to say no to requests that do not contribute to your meaningful work or will require you to work with people who negatively affect your mental health. Getting involved in national organizations, such as the Infectious Diseases Society of America and the Society of Infectious Diseases Pharmacists, provides opportunities to build a network of colleagues with diverse interests and skills. Participating in ID advocacy work, public policy, mentoring programs, and educational podcasts contributes to meaningful work. Joining international organizations, social media platforms, and publishing widens your global ID network. Engage future directors of pharmacy and ID fellowship programs in ASP because they will have the power to create change. For example, I provided travel support to South Africa for a pharmacy resident in the master’s program, knowing that his goal was to become a Director of Pharmacy. I wanted him to learn the value of global ASP research collaboration and the need for pharmacists to have protected research time.

When I transition to professor emeritus status, I will continue my global antibiotic stewardship, patient advocacy, and enthusiastically steering dental antibiotic stewardship in the United States and South Africa. Although I am concerned about the future of ASP, there is one thing I know for sure. When I turn 80, the new captains steering the antibiotic stewardship ship will be able to find me on deck wearing fuchsia pink sparkles and dancing with moves like Jagger.
